# Clinicopathological Study of Intradural Extramedullary Spinal Tumors and Its Correlation With Functional Outcome

**DOI:** 10.7759/cureus.15733

**Published:** 2021-06-18

**Authors:** Sharadendu Narayan, Shrikant V Rege, Rakesh Gupta

**Affiliations:** 1 Neurosurgery, Tata Main Hospital, Jamshedpur, IND; 2 Neurosurgery, Sri Aurobindo Institute of Medical Sciences and Postgraduate Institute, Indore, IND; 3 Neurosurgery, Mahatma Gandhi Memorial Medical College, Indore, IND; 4 Neurosurgery, Maharaja Yeshwantrao Hospital, Indore, IND

**Keywords:** denis pain scale, intradural extramedullary spine tumors, nurick grade, frankel grade, functional outcome

## Abstract

Introduction

Intradural extramedullary (IDEM) spinal cord tumors account for approximately two-thirds of largely benign intraspinal neoplasms. These are amenable to gross total excision and usually carry a good functional outcome.

Methods

In this study, we reviewed the surgical outcomes of 35 patients who underwent excision of intradural extramedullary tumors. Patient demographics, severity and duration of symptoms, and tumor characteristics (anatomical and pathological) in all operated spinal IDEM tumors were collected. The neurological findings obtained during the preoperative stage and the postoperative follow-up were evaluated according to the Frankel and Nurick grading. The back pain was assessed with help of the Denis pain scale (DPS).

Results

The histopathological outcomes of the study were as follows: six patients of neurofibroma, 12 cases of schwannoma, nine cases of meningiomas, three cases of ependymoma, one case of dorsal neurenteric cyst, two cases of epidermoid cyst, one case of cauda equina paraganglioma, and one case of filum terminale dermoid cyst. Paresthesia/numbness were the commonest symptoms (88.6%), weakness of limbs in (80%), sphincter dysfunction in 15 patients (42.9%), and paraplegia was seen in three patients (8.57%). The complications encountered were - one case each of cerebrospinal fluid (CSF) leak, surgical site infection, and pseudomeningocele. The percentage of spinal canal occupied ranged from 71-94%. The mean percentage of the spinal canal occupied by the tumor was 81.8%.

In our series, 77.14% of patients (p<0.0001) had good functional outcomes as per improvement in Frankel score. The DPS and Nurick score mean values showed a significant decrease over the follow-up duration as compared to preoperative mean values. Significant functional improvement was noted at the one-week, one-month, and one-year follow-up, with a p-value of <0.0001.

Conclusions

The IDEM tumors are usually benign and are readily detected by contrast-enhanced MRI scans. These have excellent surgical outcomes with some exceptions. Greater canal occupancy and a longer duration of symptoms are usually seen to correspond with suboptimal functional outcomes.

## Introduction

Spinal intradural extramedullary (IDEM) tumors account for two-thirds of all primary intraspinal neoplasms, but these lesions are uncommon, with a reported incidence of 3-10 per 100,000 people [1]. In the adult population, the most common IDEM tumors arise from the nerve sheath (approximately 30%) and from the meninges (approximately 25%) [2].

Over the years, there has been no significant change in the clinical symptoms and pathology of IDEM tumors. However, there have been dramatic improvements in the diagnosis and treatment with the advances of radiological and surgical techniques. Despite advances in operative techniques and neuroimaging, the morbidity associated with the resection of IDEM tumors continues to be significant [3].

The study aims to analyze patient demographics, severity and duration of symptoms, and tumor characteristics (anatomical and pathological) in all operated spinal IDEM tumors, and to correlate their preoperative neurological status and postoperative functional outcomes.

## Materials and methods

A prospective observational study was carried over a period of 1.5 years at the Department of Neurosurgery, Sri Aurobindo Institute of Medical Sciences and Postgraduate Institute, Indore, India. The study had a sample size of 35 patients of IDEM spine tumor who underwent microsurgical resection of the tumor. The Institutional Ethics Committee Sri Aurobindo Medical College and Postgraduate Institute issued approval vide no. 22.01.2014/054. Informed written consent was obtained from all patients included in the study.

Inclusion criteria

All patients undergoing surgery for solitary spinal IDEM tumors (primary/recurrent) during the study duration were included in the study.

Exclusion criteria

Patients with multiple IDEM tumors or those with illness-limiting surgical interventions were excluded from the study. Patients who were lost to follow-up or those who refused repeat MRI scans were also excluded from the study.

Methods

The registration data, duration of symptoms, and nature of complaints were recorded. Preoperative pain severity was assessed using the Denis pain scale (DPS). Preoperative functional status was recorded using the Frankel grading and the Nurick grading scales. Written and Informed consent was obtained from all patients and their representatives regarding their willingness to be a part of the study and the follow-up process. Patients underwent a contrast-enhanced MRI spine scan during the initial surgical workup. A repeat MRI scan was performed postoperatively after one month of surgery to assess the degree of excision, and also after one year of surgery to look for recurrence. The location of the tumor on sagittal and axial MRI images was recorded preoperatively.

**Figure 1:**
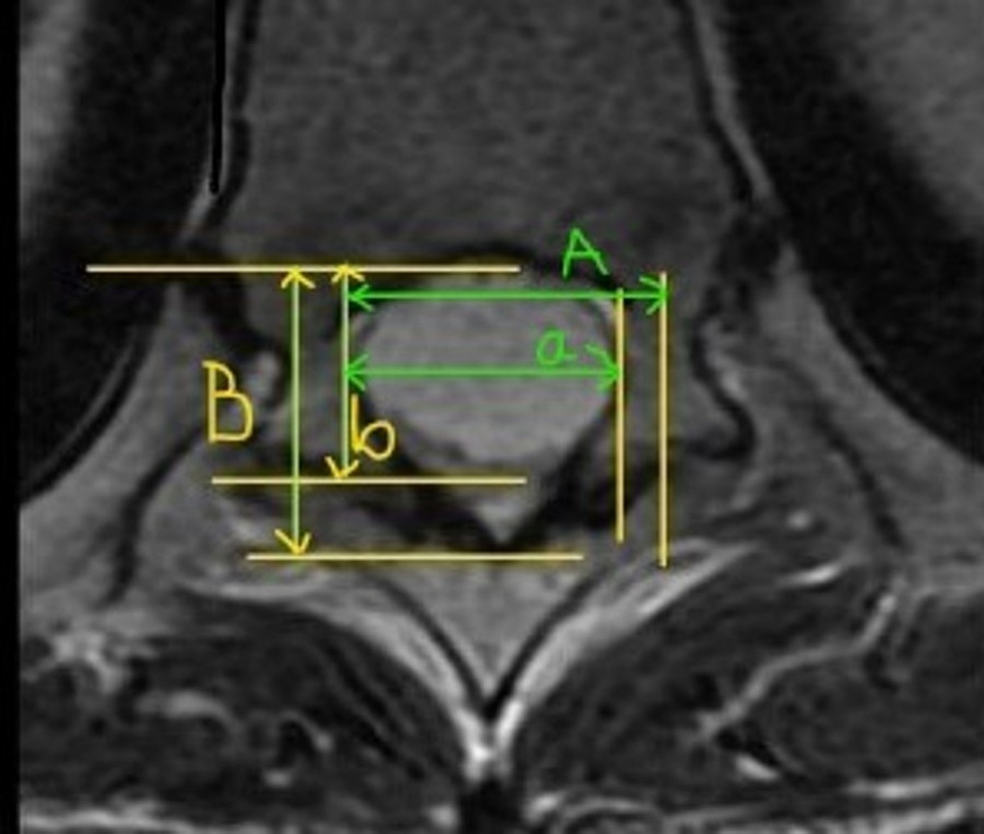
Percentage of tumor occupying intradural space was calculated with the formula {(a+b)/(A+B)} x 100 Transverse diameter of the tumor mass (a) + Longitudinal diameter of the tumor mass (b) / Transverse diameter of the intradural space (A) + Longitudinal diameter of the intradural space (B) x 100.

The percentage of tumor occupying the intradural space was calculated on the axial image showing the maximum size (Figure 1).

After relevant investigations, patients were electively posted for laminectomy and gross total excision of the tumor under general anesthesia.

Surgical technique

In this study, a microsurgical facet sparing posterior approach was used in all the cases. Thirty-three patients underwent total laminectomy at the level of the tumor, and hemilaminectomy was done in two patients. Closure of the dura mater was performed with Vicryl 4-0 round body needle in all cases. Negative pressure drainage was performed in all the cases. The drain was removed on the second postoperative day, and supported ambulation was started on the fifth postoperative day.

No intraoperative complications such as cord injury, root injury, excessive bleeding, etc. were observed.

Following surgery, the primary outcome assessment was done. Patients were graded postoperatively on the Frankel and Nurick scales at one week, one month, and one year for the evaluation of functional status. The tumor histopathology report was recorded. A postoperative MRI scan was done at one month to look for adequacy of resection.

In the secondary outcome assessment, patients were assessed for remission in pain postoperatively using the DPS at one week, one month, and one year. Complications of the surgical procedure were recorded. A repeat MRI scan was done one year postoperatively to look for recurrence.

The statistical analysis was done using Statistical Package for the Social Sciences (SPSS Statistics) version 22 (IBM Corp., Armonk, USA). Spearman correlation test, paired Student’s t-test, MANOVA, and Mann-Whitney U test were performed on the data.

We defined a "good outcome" as an improvement in the preoperative Nurick and Frankel grades. Patients with an improvement of ≥2 Frankel grade or ≥3 Nurick scores were labeled as having “significant improvement”. The patients who either had unchanged scores or showed a deterioration of Frankel or Nurick score were considered to have a “poor outcome”.

Nurick grade

Grade 0: No symptoms of spinal cord disease.

Grade I: Symptoms of spinal cord disease, but no difficulty in walking.

Grade II: Slight difficulty in walking.

Grade III: Difficulty in walking, but not so severe as to require assistance.

Grade IV: Able to walk only with another person's assistance or with the aid of a frame.

Grade V: Wheelchair- or bed-bound.

Frankel scale

A. COMPLETE: Motor and sensory loss below the lesion.

B. MOTOR USELESS: Some sensory preservation below the level of the lesion.

C. MOTOR USELESS: Motor and sensory sparing, but the patient is non-functional.

(Grade 2-3/5 Medical Research Council (MRC) scale)

D. MOTOR USEFUL: Motor and sensory sparing and the patient is functional (stands and walks).

(Grade 4/5 MRC scale)

E. RECOVERY: Complete functional recovery; reflexes may be abnormal.

Dennis pain scale

P1 No pain.

P2 Occasional minimal pain; no need for medication.

P3 Moderate pain, occasional medications, and no interruption of work or activity of daily living.

P4 Moderate pain, occasionally absent from work; significant changes in activities of daily living.

P5 Constant, severe pain; chronic pain medications.

## Results

Preoperative symptoms

**Figure 2:**
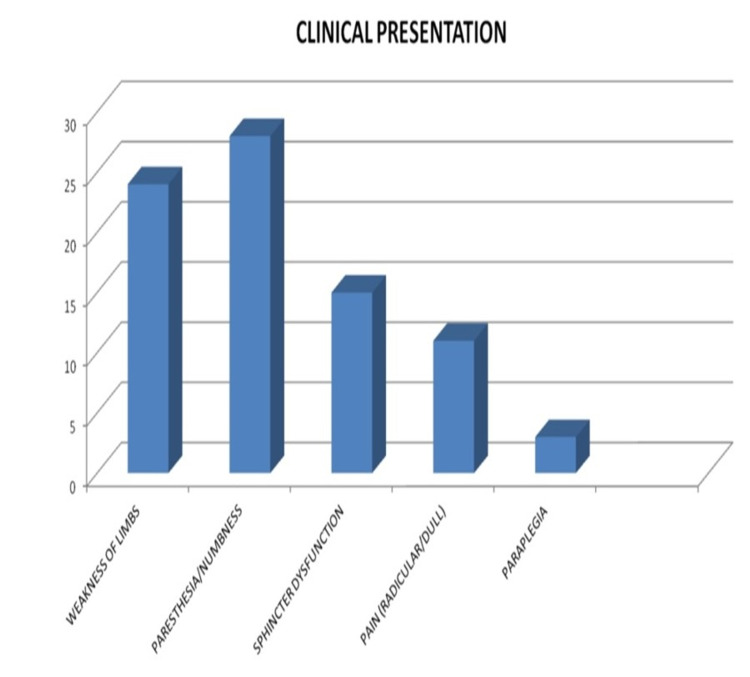
Patients' presenting symptoms

The most common preoperative complaint was paresthesia/numbness in 88.6% of patients, followed by weakness of limbs in 80%. Sphincter dysfunction was present in 15 patients (42.9%), dull aching/radicular pain was seen in 31.4% (11/35) of patients. Paraplegia was seen in three patients (8.57%) (Figure 2).

Thirteen patients had a duration of symptoms less than six months; the remaining 22 had a duration of symptoms more than months. The mean duration of symptoms was 6.4 months. Good functional outcome was seen in all 13 patients with a duration of symptoms less than six months, and in 14 of the patients, with symptoms lasting more than six months (p=0.013). 

Distribution of tumors

**Figure 3:**
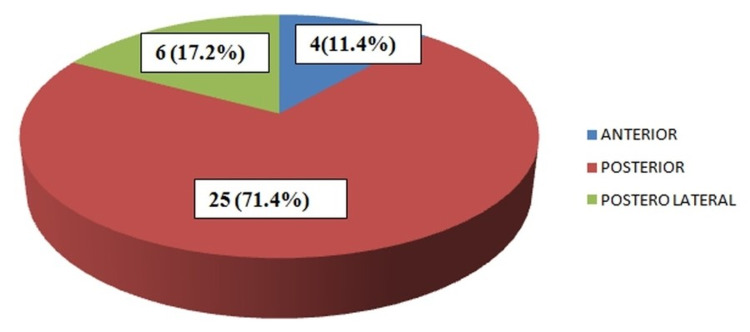
Axial tumor distribution

The axial distribution in the spinal cord is presented in Figure 3.

The sagittal distribution of tumors is presented in Table 1.

**Table 1:** Tumor sagittal location (n=35)

Location	Nerve Sheath Tumor	Meningioma	Ependymoma	Others	Percentage (%)
Cervical	6	2	0	0	22.8
Thoracic	8	6	0	3	48.6
Dorsol umbar	2	1	2	0	14.3
Lumbar	2	0	1	2	14.3

**Figure 4:**
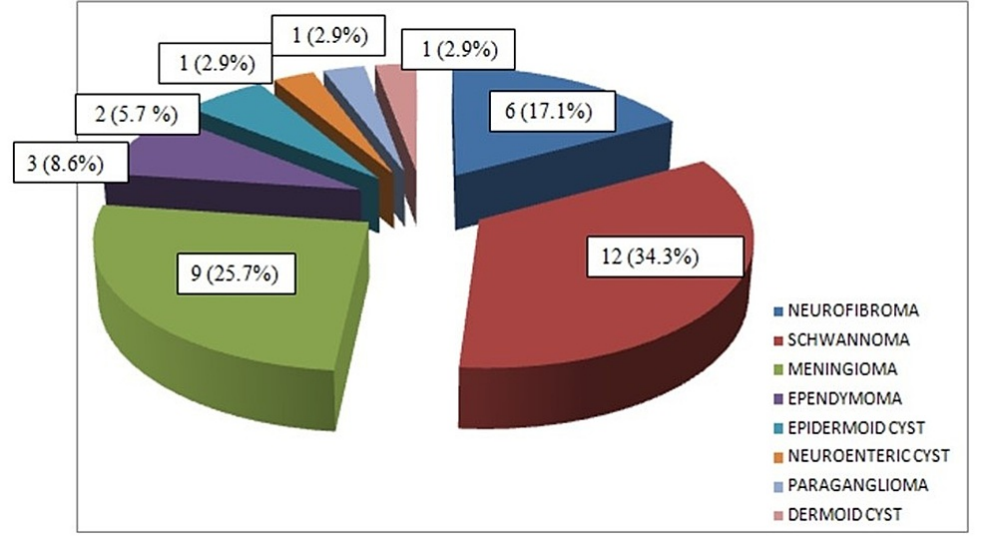
Pathological tumor types

The pathological tumor types as recorded are presented in Figure 4.

**Figure 5:**
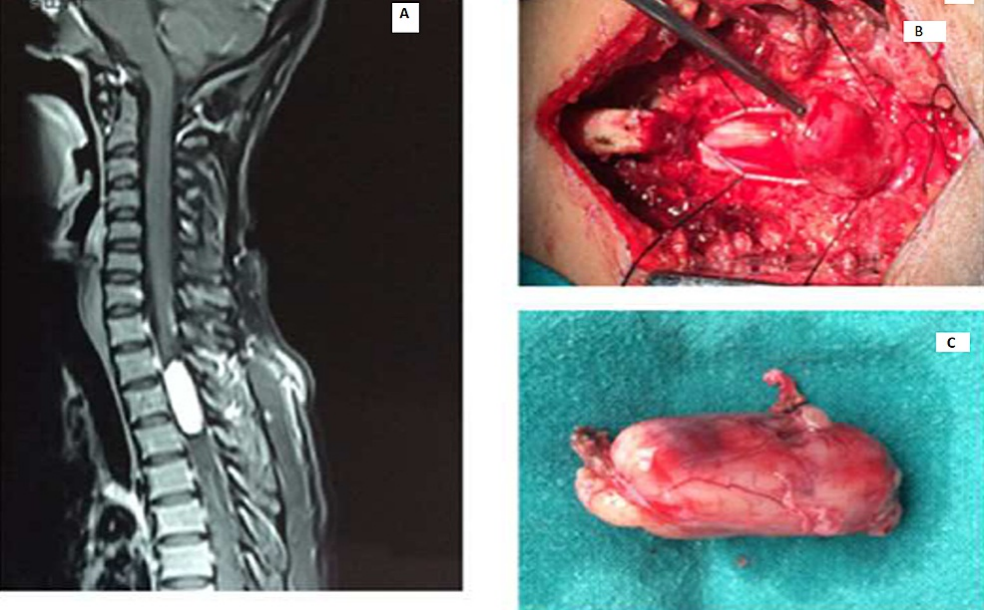
Cervical neurofibroma A: Contrast-enhanced sagittal MRI cervical spine with tumor. B: Intraoperative image with laminectomy done and tumor being dissected. C: Tumor specimen.

Nerve sheath tumor was the most common type. There were six patients with neurofibroma (Figure 5) and 12 patients with schwannoma as the histopathological diagnosis. All six cases of neurofibroma were seen in males, whereas the schwannomas group comprised nine female and three male patients.

**Figure 6:**
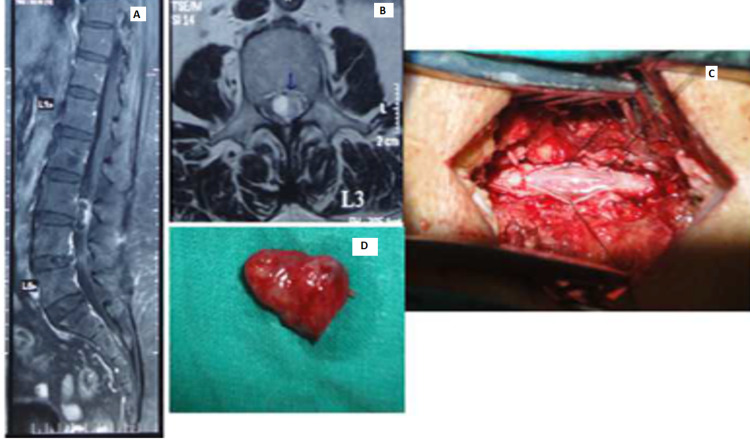
Lumbar spine ependymoma A: MRI post-contrast image, sagittal section with tumor having patchy peripheral enhancement. B: MRI post-contrast image, axial cross-section. C: Intraoperative image. D: Tumor specimen.

There were nine cases diagnosed as meningiomas - eight female and one male. There were three cases of ependymoma (Figure 6); both cases were seen in males.

**Figure 7:**
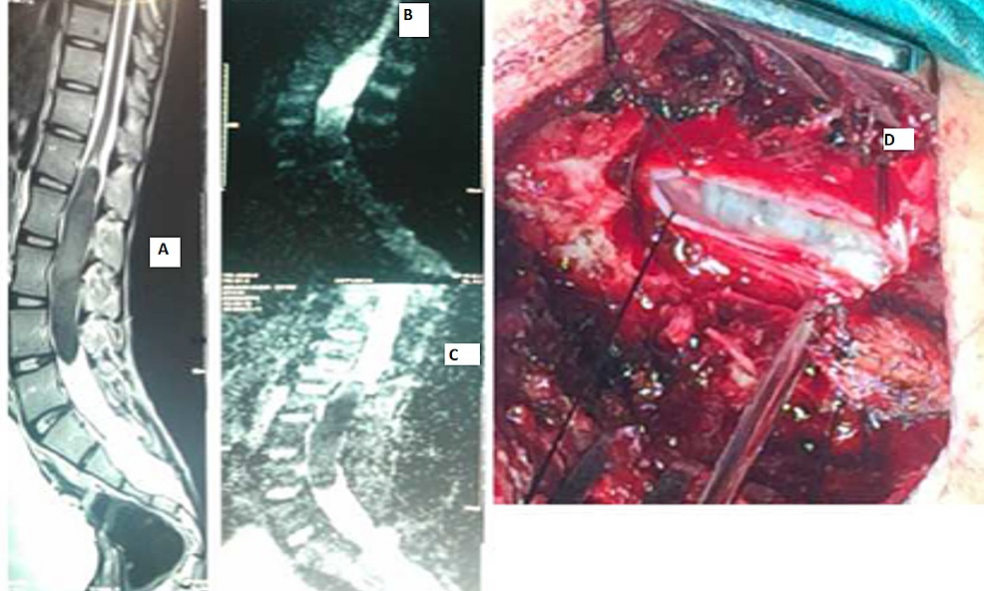
Lumbar epidermoid cyst A: MRI lumbosacral spine sagittal image with hypointense tumor mass on post contrast image. B: MRI lumbosacral spine with restriction on Apparent Diffusion Coefficient sequence. C: Diffusion-weighted image showing restriction. D: Intraoperative pearly white appearance of the cyst.

There was one case of neuroenteric cyst seen at the thoracic level, which was noted in the female patient. There were two cases of epidermoid cyst (Figure 7).

**Figure 8:**
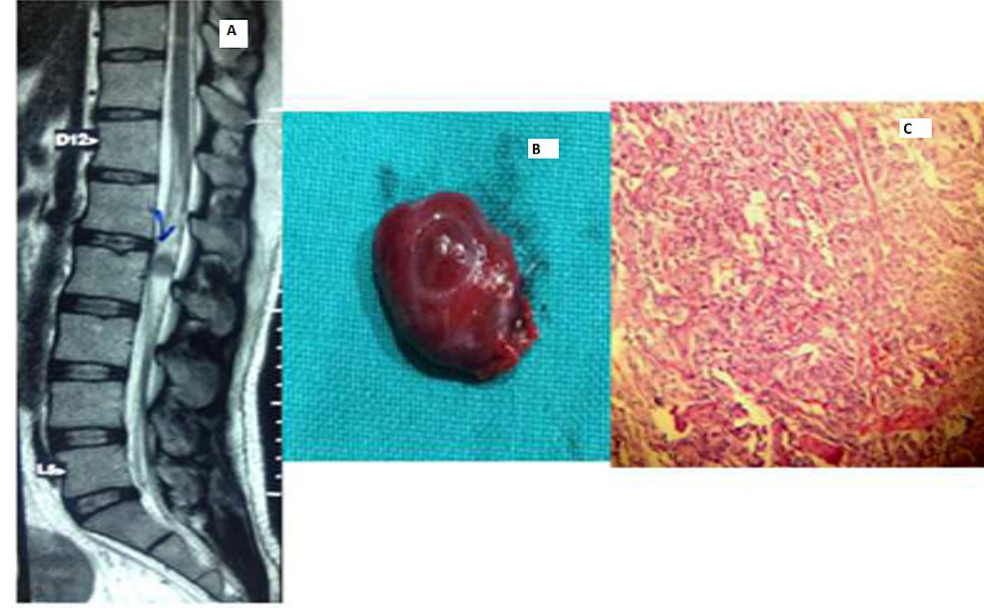
Paraganglioma of cauda equina region A: MRI T2-weighted sagittal image with hypo intense lesion. B: Cauda equina paraganglioma sample after excision. C: Strong synaptophysin expression in tumor cells.

There was one case of paraganglioma of the cauda equina region seen in a male patient (Figure 8).

**Figure 9:**
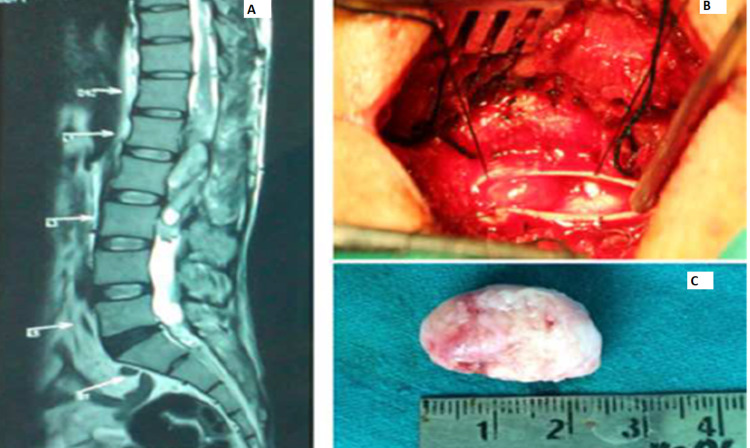
Filum terminale dermoid cyst A: MRI T2-weighted sagittal image with iso- to hypointense image. B: Intraoperative image. C: Tumor specimen with stratified keratin and hair.

One case of filum terminale dermoid cyst was noted in a male patient (Figure 9).

Canal occupancy

The percentage of spinal canal occupied ranged from 71-94%. The least percentage occupied was seen in a case of thoracic posterolateral neurofibroma (71%). The maximum percentage occupied was in a case of anteriorly placed cervical meningiomas (94%). The mean percentage of the spinal canal occupied by the tumor was 81.8%.

Follow-up duration

Patients were followed up for a mean duration of 16 months with a range of 13-20 months.

Surgical treatment

The tumors located posterior or dorsal to the cord (25/35; 71.4%) were resected with a posterior approach with laminectomy. The posterolateral located tumors (7/35; 20%) and the anteriorly situated tumors (3/35; 8.6%) also underwent gross total excision with gentle cord rotation and traction via a posterior approach.

Thirty-three patients (94.3%) underwent total laminectomy and microsurgical excision of the tumor. Two patients (2/35; 5.7%) underwent a hemilaminectomy and microsurgical excision of the tumor, one of these patients was a case of a dermoid cyst at lumbar level and the other patient was a case of an epidermoid cyst at the thoracic level.

Thirty-four patients (34/35; 97.1%) underwent microsurgical gross total excision of the tumor. However, one patient with recurrent thoracolumbar ependymoma underwent subtotal excision of tumor 2.9% (one out of 35 patients) due to severe adhesions with conus medullaris.

Nine out of 35 (25.7%) meningioma cases underwent a Simpson grade 2 resection. 

Morbidity and mortality

The complications encountered in the postoperative period were - CSF leak from wound site in a case of thoracic neurofibroma, surgical site infection in one case of lumbar paraganglioma, and pseudomeningocele in a case of lumbar dermoid cyst. There was no incidence of spinal instability, meningitis, or operative mortality.

Functional outcome

At the end of one year, follow-up notes were available for 35 patients with intradural extramedullary tumors. Patients were followed up for a mean duration of 16 ± 2.1 months.

In our series 27/35 (77.14%) patients had good outcomes as per improvement in Frankel score at the time of last follow up, and 2/35 (5.71%) patients had improvement of ≥ 2 grades on Frankel score and were said to have significant improvement. However, eight (22.9 %) patients had no improvement in postoperative Frankel grade and had poor outcomes.

In our series, 35/35 patients had an improvement in the Nurick grade. Out of these, 11 patients had an improvement of single Nurick grade at the last examination, and 22/35 (62.8%) patients had an improvement of ≥ 2 grades on the Nurick scale. There were 4 (11.4%) patients with significant improvement, with an improvement of ≥ 3 grades as per Nurick Grade, and 29/35 (82.86 %) patients were ambulatory without support at the last follow-up.

**Figure 10:**
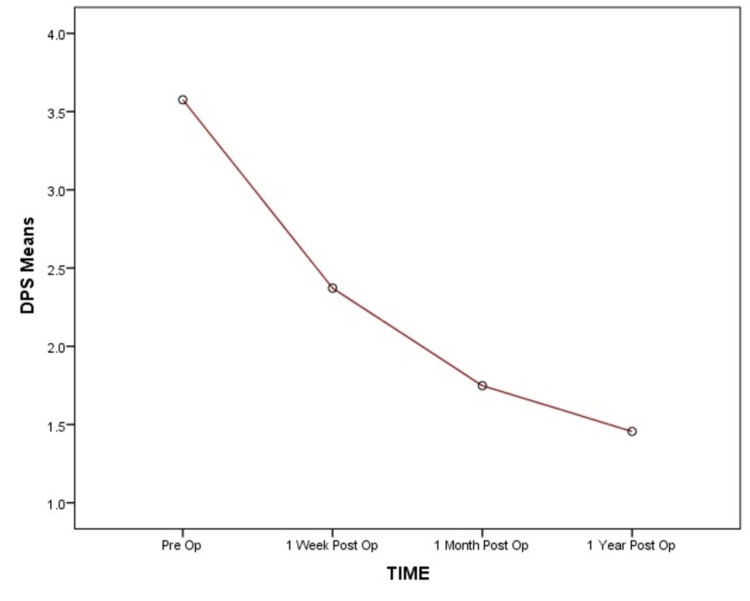
Postoperative Denis pain scale The mean values of Denis pain scale show a significant decrease at one week, one month, and one year postoperative interval.

The Denis pain scale and Nurick score mean values show a significant decrease over the follow-up duration as compared to preoperative mean values (Figure 10). 

Significant functional improvement was noted at one-week, one-month, and one-year follow-up with a p-value of <0.0001 as per the MANOVA test (Table 2).

**Table 2:** Clinical improvement

	Pre-Op	1 week	1 month	Last follow-up	p-value
Denis pain scale	3.57 ± 0.88	2.37 ± 0.54	1.74 ± 0.65	1.45 ± 0.51	<0.0001
Nurick grade	3.94 ± 1.21	3.0 ± 0.84	2.17 ± 1.12	1.97 ± 1.07	<0.0001

The factors affecting the functional outcome are detailed in Table 3.

**Table 3:** Factors affecting the functional outcome

Parameter	No. of patients	Good outcome	Poor outcome	P-value
Age (Mean 41.8 years)				0.443
≤40	18	15	3	
>40	17	12	5	
Gender				0.004
Male	16	16	0	
Female	19	11	8	
Pre-Op Neurological Status				
Frankel Grade A/B/C	19	13	6	0.244
Frankel Grade D/E	16	14	2	
Pre-Op duration of symptoms (Mean duration 6.4 months)				
≤ 6 months	13	13	0	0.013
> 6 months	22	14	8	
Axial Tumor location				
Anterior	4	3	1	0.779
Posterior	25	20	5	
Postero-lateral	6	4	2	
Sagittal location of Tumor				
Cervical	8	7	1	0.083
Thoracic	17	10	7	
Thoraco-lumbar	5	5	0	
Lumbar	5	5	0	
Spinal canal occupied by tumor in % (Mean 81.7%)				
≤ 80%	15	15	0	0.006
>80%	20	12	8	
Surgical approach				
Total laminectomy	33	25	8	0.427
Hemilaminectomy	2	2	0	

## Discussion

Intradural extramedullary tumors are usually benign and are seen to account for two-thirds of primary spinal tumors. Studies indicate that five females and three males out of 1,000,000 people are affected by primary spinal tumors each year [4]. As per Nittner [5] the incidence of IDEM tumors is 0.3 out of 100,000 people. The clinical presentation and the surgical outcome are influenced by the anatomical tumor location.

A contrast-enhanced MRI scan is the investigation of choice. The IDEM generally appears isointense on T1-weighted images, hyperintense on T2-weighted images, and shows enhancement with gadolinium contrast. Plain radiographs and CT scans show vertebral body involvement, pedicle erosion, and widened neural foramina.

In our study, we analyzed the patient demographical data, the symptoms, tumor profile, functional outcome, complications, and recurrence in 35 patients. The age distribution in our study showed a peak incidence at 21-30 years. This was unlike the age group reported by Seppala et al. [6] and Fernandes et al. [7], where the commonest incidence was between 40-60 years of age.

The gender distribution in our study was 45.75% male and 54.3% females. This was comparable to rates reported by Seppala et al. [6], Fernandes et al. [7], Song et al [2], and Nittner [5] where the incidence was equal in males and females. There was a significant poor functional outcome noted in the female gender (Table 3). This is, however, a confounding variable as this group of patients also had other associated factors, such as prolonged duration of more than six months, contributing to poor functional outcomes.

As per Nittner [5], more than 50% of IDEM tumors were in the thoracic region, with 22% incidence in cervical and 22% incidence at the lumbosacral level. In our study, the rates were comparable, with 48.6% of tumors occurring at the thoracic location, followed by 22.8% in cervical and 14.3% at the thoracolumbar and lumbosacral locations.

In our study, nerve sheath tumors formed the largest histopathological group with 18 (51.4%) patients, followed by nine (25.7%) patients of meningiomas, and the third most common group of three (8.6%) patients with filum terminale ependymomas. The histopathological distribution of IDEM tumors in our study conformed to observations made by Arora and Kumar [8], Song et al. [2], Kankane et al. [9], and Ahn et al. [1]. The age of patients and histopathology of the tumor were not seen to affect the functional outcome in our study. This was as seen in the study by Arora and Kumar [8].

It is conventionally thought that the longer preoperative duration of symptoms, severe preoperative neurological deficits, and proximal or anterior location of the tumor contribute to poor prognosis [6]. 

However, Ahn et al. [1] and Song et al. [2] in their studies found no relation between the degree and duration of preoperative symptoms with the postoperative functional outcome. This was unlike our study wherein patients with longer preoperative duration of symptoms were seen to have poorer functional outcomes, p=0.013 (Table 3). Arora and Kumar [8] in their study observed similar findings. 

Klekamp and Samii [10] have reported favorable outcomes when the interval from diagnosis to surgery was short, prior to the development of severe neurological deficits. Similar findings were observed in our study.

In our study, there was no association of the sagittal and axial tumor location with the functional outcome. This was unlike the literature where the thoracic and ventral locations of tumor were noted to have poor postoperative outcomes [11]. 

The percentage of the tumor occupying the spinal canal had a significant correlation with the functional outcome. The percentage of spinal canal occupancy by tumor was inversely related to the functional outcome, p=0.006 (Table 3). 

In our study, there was a significant decrease in the DPS and Nurick score observed over the follow-up period. The p-value was 0.001 and was suggestive of a significant decrease in the pain symptoms postoperatively and a significant improvement in the functional outcome as per the Nurick scale postoperatively. The significant increase in patient functional outcome was early as one week postoperatively (Table 3). 

The surgical technique adopted in our study did not statistically affect the outcome. However, it must be kept in mind that minimally invasive surgical techniques are seen to have better functional outcomes in trained hands. In our study, owing to a lack of resources and technical expertise, the posterior microsurgical approach was adopted in all patients

*Complications: *CSF leak from the wound site was seen in a case of thoracic neurofibroma which was managed successfully with lumbar drain insertion and acetazolamide tablets. Surgical site infection was noted in one case of lumbar paraganglioma which was managed with oral antibiotics. A case of lumbar dermoid cyst developed pseudomeningocele after three weeks of surgery. The patient was taken up for re-exploration and the dural defect was repaired with a fascia graft with fibrin glue reinforcement.

The postoperative recurrence rate of intradural extramedullary spinal cord tumors varied between 5% and 16% in the literature [12]. There was no recurrence observed at the last follow-up in our study; however, the mean follow-up duration of 16 months is inadequate to comment on the same.

## Conclusions

Intradural extramedullary tumors are largely benign in nature and form the largest group of primary intraspinal tumors. They are easily visualized on contrast-enhanced MRI scans. These tumors are amenable to microsurgical gross total resection and generally have a good functional outcome. It is observed that greater canal occupancy and a longer duration of symptoms correspond with poor functional outcomes.
